# Beyond Single Enzymes: System-Level Fungal Transformation of Halogenated Nitrophenols

**DOI:** 10.3390/jof12070493

**Published:** 2026-07-04

**Authors:** Gerardo Aguilar, Christian Krohn, Alexis Marshall, Sali Khair Biek, Julie A. Besedin, Courtney Pilcher, Attila Tottszer, Leadin S. Khudur, Andrew S. Ball

**Affiliations:** 1 Department of Biology, School of Science, RMIT University, Melbourne, VIC 3083, Australia; christian.krohn@rmit.edu.au (C.K.); s3681949@student.rmit.edu.au (S.K.B.); andy.ball@rmit.edu.au (A.S.B.); 2 Solving Plastic Waste CRC, RMIT University, Bundoora, VIC 3083, Australia; 3 ALS Environmental Smithfield, NSW, Australia

**Keywords:** fungi, biotransformation, biological transformation, bioremediation, organic contaminants, environmental pollution, metabolic pathway, system-level process

## Abstract

Despite increasing interest in fungal remediation systems for the treatment of persistent contaminants, the mechanisms governing fungal transformation of halogenated organic compounds remain poorly resolved. The aim of this study was to determine whether the transformation of halogenated nitrophenols is driven by isolated extracellular enzymes and cofactor-dependent oxidative activity or instead reflects coordinated system-level fungal metabolism. To address this question, we investigated the transformation of 2-chloro-4-nitrophenol (2C4NP) and 5-fluoro-2-nitrophenol (5F2NP) by ascomycete fungi *Caldariomyces fumago* (*C. fumago*) and *Curvularia* sp. under varying nutrient and cofactor conditions. Whole-culture transformation, crude supernatant activity, purified enzyme assays, intracellular detoxification responses, and genome-resolved functional annotation were integrated to evaluate the relative contributions of extracellular and intracellular processes. Transformation was strongly dependent on fungal species, substrate identity, nutrient availability, and cofactor composition. *C. fumago* achieved complete transformation of 2C4NP and up to 85.3% transformation of 5F2NP, whereas *Curvularia* sp. exhibited strict Na_3_VO_4_-dependent transformation of 5F2NP. Crude supernatants retained partial transformation capacity, achieving ~40–45% substrate depletion under conditions supporting whole-culture activity. Purified chloroperoxidase and laccase showed negligible independent activity and did not reproduce whole-culture transformation behavior. Lignin peroxidase activity was consistently induced during contaminant exposure and peaked during periods of maximum transformation. Cytochrome P450 inhibition did not prevent transformation. Baseline glutathione S-transferase activity was detected in both fungi, and comparative genome analysis identified conserved intracellular detoxification-associated enzyme alongside divergent extracellular oxidative enzyme repertoires. Together, these findings demonstrate that transformation of halogenated nitrophenols by fungi cannot be explained by isolated extracellular enzymes alone but is consistent with coordinated extracellular and intracellular system-level metabolism. These findings highlight an underexplored role for integrated fungal metabolic systems in bioremediation and provide a mechanistic basis for developing a scalable fungal platform for treatment of persistent halogenated contaminants.

## 1. Introduction

Halogenated organic compounds constitute a major class of environmental contaminants due to their widespread use in industrial processes and agriculture, and their persistence in natural systems [1]. Their resistance to transformation in the environment is largely governed by the strength of the carbon–halogen bond, particularly in fluorinated compounds, which can exceed 100 kcal mol^−1^, limiting both abiotic breakdown and biological transformation [2,3]. Halogenated nitrophenols represent an environmentally relevant subclass of these contaminants. They are introduced into the environment through pesticide application and industrial discharge and are routinely detected in soil and water systems [4]. These compounds have been reported at concentrations up to 44.1 μg L^−1^ in surface waters and applications rates of up to 312 g ha^−1^ in agricultural setting [2,5,6]. Beyond persistence, their combined nitro and halogen substituents increase electrophilicity and toxicity towards microbial systems including fungi [7,8]. This combination of environmental relevance and biological stress makes these compounds suitable for investigating fungal transformation mechanism under conditions where contaminant transformation persist despite limited fungal growth and restricted nutrient availability [8].

Fungal transformation of xenobiotics has traditionally been attributed to extracellular oxidative enzymes, particularly laccases and peroxidases from ligninolytic basidiomycetes such as *Phanerochaete chrysosporium* and *Trametes versicolor* [9,10]. These systems have demonstrated the ability to oxidize phenolic and halogenated compounds, including chlorophenols and related aromatic substrates, often achieving high removal efficiencies under optimized conditions [4,11]. However, these transformations frequently depend on mediator compounds, enzyme cofactors complex enzyme mixtures, and tightly controlled redox environments. As a result, purified enzymes often show reduced or incomplete transformation when removed from their native biological context. For example, purified laccase systems showed only partial transformation of highly chlorinated substrates such as pentachlorophenol [12], while immobilized laccase systems exhibited substantial reduction in catalytic efficiency and substrate range [13].

Despite extensive biochemical characterizations, translation of these enzyme systems into commercial or field-scale applications has remained limited. While laccases have been explored in wastewater treatment, textile dye decolorization, and pulp processing, their deployment typically requires immobilization, mediator addition, or continuous enzyme replenishment, increasing system complexity and operational cost [12,13]. For haloperoxidases, including chloroperoxidase from *Caldariomyces fumago*, industrial applications have largely focused on fine chemical synthesis rather than environmental remediation, reflecting challenges in maintaining catalytic activity and stability under environmental conditions [14]. This highlights a broader gap between enzymatic capability and scalable application.

At the organism level, however, field-based applications have been reported. For example, *Trametes versicolor* has been applied in engineered soil systems for the treatment of pentachlorophenol, reducing concentrations from 800–1000 mg kg^−1^ to as low as 0–9.94 mg kg^−1^ over 2.5 years under low maintenance conditions. These systems rely on intact fungal biomass with lignocellulosic matrices and passive aeration, rather than isolated enzyme deployment [15]. In contrast to these basidiomycete systems, ascomycete fungi such as *C. fumago* and *Curvularia* spp. produce haloperoxidases with direct relevance to halogenated substrate chemistry. Chloroperoxidases from *C. fumago* can catalyze halogenation, oxygen transfer, and oxidative dehalogenation reactions [16,17,18]. *Curvularia* sp. produce vanadium-dependent haloperoxidases that exhibit high catalytic stability and distinct cofactor requirements [19,20]. The catalytic activity of fungal haloperoxidases is strongly influenced by cofactor availability. Heme-dependent choloroperoxidases require iron for synthesis and catalytic function, whereas vanadium-dependent haloperoxidases require incorporation of vanadate to form an active enzyme [19,20]. Consequently, cofactor availability may directly influence contaminant transformation capacity and provide a mechanistic basis for evaluating the relative contributions of enzyme-specific activity and broader system-level responses. Notably, vanadium-dependent haloperoxidases operate via a catalytic mechanism in which the vanadate cofactor remains in a stable oxidation state, distinguishing them from heme-dependent systems that rely on redox cycling during catalysis [20]. Despite these favorable proprieties, there is limited evidence that these enzymes alone can reproduce the transformation behavior observed in intact fungal cultures.

Previous studies have shown that *C. fumago* and *Curvularia* sp. can transform halogenated nitrophenols under defined media conditions, including at concentrations that inhibit fungal growth. Transformation occurred under apparent stationary-phase conditions, where little to no measurable biomass increase was observed, indicating that substrate removal was not directly linked to growth-associated metabolism [1]. These observations suggest that transformation is mediated by oxidative and detoxification processes operating within the intact fungal system rather than through conventional metabolic utilization of the contaminant. Furthermore, the persistence of transformation under growth-constraining conditions suggests that environmental factors, including nutrient availability and cofactor composition, may regulate oxidative and detoxification processes within the intact fungal systems.

However, these observations raise a central mechanistic question: does transformation arise primarily from individual extracellular enzymes and cofactor-dependent oxidative activity, or from coordinated interactions within the intact fungal system? This distinction has direct implications for the development of fungal remediation technologies. Enzyme-based approaches assume that catalytic function can be isolated and deployed independently. Yet, fungal oxidative transformations often depend on coupled redox processes and coordinated extracellular and intracellular activities that may not be preserved outside of the native biological context. Despite extensive characterization of fungal oxidative enzymes, their contribution to whole system transformation remains largely inferred rather than experimentally resolved [9,21].

The aim of this study was to determine whether transformation of halogenated nitrophenols by *C. fumago* and *Curvularia* sp. could be explained by isolated extracellular enzymes and cofactor-dependent oxidative activity or instead reflected coordinated systems-level fungal metabolism. This was addressed through direct comparison of whole-culture transformation, crude extracellular supernatant activity, purified enzyme assays, intracellular detoxification responses, and genome-resolved functional annotations. By resolving the relative contributions of extracellular and intracellular processes, this work establishes a mechanistic framework for evaluating enzyme-based versus whole-organism fungal remediation strategies for halogenated contaminants.

## 2. Materials and Methods

### 2.1. Fungal Strains and Products

*C. fumago* ICMP5613 was purchased from Land Care Research New Zealand. *Curvularia* sp. was provided by the University of Queensland. Fungal cultures were maintained using sterile water storage at 25 °C. Tap water was autoclaved at 121 °C for 15 min and used to preserve mycelial fragments in sterile containers, following the established methods for long-term fungal storage [22]. For routine culturing, strains were grown on potato dextrose agar (PDA) (Merck, Truganina, VIC, Australia) [1]. Media were prepared according to the manufacturer’s instructions and autoclaved at 121 °C, for 15 min at 15 psi. For plate inoculation, sterile agar plugs were used to aseptically transfer mycelium samples to the center of a 90 mm Petri plates. Inoculated plates were incubated at 25 °C ± 2 °C. Unless otherwise stated, all chemicals were sourced from Merck (Truganina, VIC, Australia).

### 2.2. Media Composition and Cofactor Modification

A defined glucose-salts media based on Pickard [23] was used as the base medium for *C. fumago* and *Curvularia* sp. The base medium (1 L) consisted of glucose (40 g, C_6_H_12_O_6_), malt extract (20 g), sodium nitrate (2 g, NaNO_3_), potassium chloride (2 g, KCl), potassium dihydrogen phosphate (2 g, KH_2_PO_4_), magnesium sulfate heptahydrate (1 g, MgSO_4_•7H_2_O), and ferrous sulfate monohydrate (20 mg, FeSO_4_•H_2_O), prepared in Mill-Q water. Following pH adjustment to 6.5, media was autoclaved at 121 °C, for 15 min at 15 psi. To evaluate the effect of cofactor availability on transformation, media were supplemented with either iron (Fe^2+^) or sodium orthovanadate (Na_3_VO_4_). Iron was supplied as FeSO_4_•H_2_O in the base medium, while Na_3_VO_4_ was added to a final concentration of 10 μM, where indicated.

Media Preparation and Systematic Glucose Reduction

All media were prepared using Milli-Q water. A sterile 50% (w/v) glucose stock solution was prepared by filtration (0.22 μm) and added aseptically to autoclaved salts media following cooling to room temperature. To assess the influence of nutrient availability on contaminant transformation, the defined bae medium was uniformly scaled to 75%, 50%, and 25% of the standard formulation (40 g L^−1^) [24], corresponding to final glucose concentrations of 30, 20, and 10 g L^−1^, respectively (Table 1). All other medium components were reduced proportionally to maintain consistent nutrient ratios across conditions.

To evaluate the role of metal cofactors, *C. fumago* and *Curvularia* sp. were initially cultured in the Fe-containing base medium (FeSO_4_•H_2_O). For vanadate-associated conditions, FeSO_4_•H_2_O was omitted from the medium and sodium orthovanadate (Na_3_VO_4_) was added to a final concentration of 10 μM. This enabled direct comparison of *Curvularia* sp. under Fe-containing (heme-permissive) and Fe-omitted, vanadate-supplemented conditions.

### 2.3. Transformation Experiments Using Halogenated Nitrophenols

#### 2.3.1. Substrate Preparation

Stock solutions (0.3 M) of 2-chloro-4-nitrophenol (2C4NP) and 5-fluoro-2-nitrophenol (5F2NP) were prepared in methanol, filter-sterilized (0.22 μm), and added aseptically to autoclaved media.

#### 2.3.2. Experimental Design and Transformation Assays

Transformation experiments were conducted under the conditions outlined in Table 1, using a defined base medium with systematic variation in cofactor availability (Fe^2+^ or Na_3_VO_4_) and carbon concentration (40, 30, 20, and 10 g L^−1^ glucose). Both *C. fumago* and *Curvularia* sp. were evaluated in Fe^2+^ containing base medium, while *Curvularia* sp. was additionally assessed in Fe-omitted, Na_3_VO_4_-supplemented medium (10 μM), allowing direct comparison between cofactor conditions. Cultures were amended with 0.3 mM and 1 mM 2C4NP, or 9 mM 5F2NP, as previously described [1]. The higher concentration of 5F2NP was selected to ensure sufficient analytical signal relative to background media absorbance. Experiments were conducted in 100 mL liquid medium and incubated at 27 °C ± 2 °C with agitation at 120 rpm. All experimental conditions were conducted in triplicate (*n* = 3). To assess the contribution of cytochrome P450-mediated oxidation, parallel experiments were conducted in the absence and in the presence of 1 mM 1-aminobenzotriazole (ABT), a known P450 inhibitor [11]. ABT was added aseptically to cultures prior to contaminant addition, and transformation kinetics were compared between inhibitor-treated and untreated conditions.

#### 2.3.3. Transformation of Halogenated Nitrophenols

Changes in apparent parent compound concentration were monitored using a Shimadzu UV-1800 spectrophotometer (Kyoto, Japan) at 420 nm, based on loss of absorbance associated with the nitrophenol chromophore [1]. Calibration curves were generated over the range of 0.001–0.3 mM and demonstrating high linearity, R^2^ = 0.996–0.998. Samples exceeding the linear detection ranges were diluted (1:6, *v*/*v*) with assay buffer prior to analysis. Temporal changes in compound concentrations were measured at 0, 8, 24, 56, and 72 h following inoculation.

#### 2.3.4. Screening for Transformation Products

In selected experiments, culture supernatants collected during the observed depletion of 2C4NP or 5F2NP were additionally screened for low-molecular-weight transformation products using gas chromatography–mass spectrometry (GC-MS) and thin-layer chromatography (TLC) under standard analytical conditions [25].

### 2.4. Enzyme Assays

#### 2.4.1. Crude Enzyme Preparation

Fungal strains were cultured under the conditions described above. Crude extracellular enzyme fractions were obtained directly from culture supernatants (10,000× *g* for 10 min at 20 °C). The supernatant was collected without further purification. Supernatants were analyzed immediately. Supernatants were collected at 24 h intervals.

#### 2.4.2. Direct Transformation of Halogenated Nitrophenols by Crude Culture Supernatants

Cell-free supernatants were evaluated for their ability to directly transform halogenated nitrophenols in the absence of fungal biomass. Crude culture supernatants were obtained as described in Section 2.4.1 and immediately used without further purification. Reaction mixtures (200 μL total volume) contained crude extracellular enzymes and substrate (2C4NP or 5F2NP) at the same concentrations used in whole-culture experiments. Reactions were conducted at 25 °C in a microplate reader, and changes in substrate concentration were monitored spectrophotometrically at 420 nm over 60 min. Measurements were taken at 5 min time points to capture initial transformation kinetics. Control reactions included: (i) supernatant-free controls to assess abiotic transformation, (ii) heat-denatured supernatants (100 °C, 20 min) used as matrix-matched controls (50 μL in 200 μL; 25% *v*/*v*) to account for enzyme-independent effects, including dilution and background absorbance. All assays were performed in triplicate (*n* = 3).

#### 2.4.3. Chloroperoxidase (CPO) and Vanadium-Dependent Chloroperoxidase (VCPO) Activity

Chloroperoxidase (CPO) and vanadium-dependent chloroperoxidase (VCPO) activities were quantified using a potassium iodide/hydrogen peroxide (KI/H_2_O_2_) assay. Reaction mixtures (3 mL final volume) contained potassium phosphate buffer (pH 2.75), 12 μmol KI, and 2 μmol H_2_O_2_ at 25 °C. Crude culture supernatant was added at defined volumes [26]. Iodide oxidation was monitored as the linear increase in absorbance at 350 nm. Reaction rates were calculated from the initial linear region of the A_350_ trace and expressed as ΔA_350_ min^−1^. As both CPO and VCCPO generate free iodine via peroxide-dependent halide oxidation this assay enables direct comparison of iodide-oxidizing activity under identical conditions [20].

#### 2.4.4. Laccase Activity Assay

Laccase activity was determined spectrophotometrically at 25 °C using 2 mM 2,2′-azino-bis (3-ethylbenzothiazoline-6-sulfonic acid) (ABTS) in 100 mM phosphate-citrate buffer (pH 4.0). Reactions were performed in 3 mL quartz cuvettes (1 cm path length) [27]. Oxidation of ABTS to the cation radical ATBS•^+^ was monitored as the increase in absorbance at 420 nm. Initial reaction rates were calculated from the linear region of the absorbance trace (0–60 s). Calibration using purified laccase (Sigma-Aldrich, Truganina, VIC, Australia) 0–0.2 U per reaction demonstrated linearity between enzyme units and initial rate (ΔA_420_ min^−1^), described by: ΔA_420_ min^−1^ = 0.0833 × U (R^2^ = 0.991). All activities were calculated from measurements within the validated linear range.

#### 2.4.5. Manganese Peroxidase (MnP) Activity Assay

Manganese peroxidase (MnP) activity was measured using phenol red oxidation assays in 200 μL microplate format [28]. Reaction mixtures contained 50 mM sodium malonate buffer (pH 4.5), phenol red, MnSO_4_, and crude culture supernatant. Reactions were initiated by the addition of H_2_O_2_. Oxidation of phenol red was monitored as the increase in absorbance at 610 nm. Measurements were recorded at 30 s intervals over 3 min using a microplate reader (Polarstar Omega, BMG Labtech, Mornington, VIC, Australia). Enzyme activity was calculated from the linear portion of the absorbance trace following correction with enzyme free controls [29,30].

#### 2.4.6. Lignin Peroxidase (LiP) Activity

Lignin peroxidase (LiP) activity was determined by monitoring the oxidation of veratryl alcohol to veratraldehye at 310 nm in a 200 μL microplate format [31]. Reaction mixtures contained 50 mM sodium tartrate buffer (pH 3.0), veratryl alcohol (final concentration ~2mM), and crude culture supernatant. Reactions were initiated by the addition of H_2_O_2_. Product formation was monitored as the increase in absorbance at 310 nm. Measurements were recorded at 30 s intervals over 3 min using a microplate reader. Enzyme activity was calculated from the linear portion of the absorbance trace following subtraction of enzyme-free controls.

#### 2.4.7. Quantification of Extracellular Protein

Total extracellular protein was quantified using the Qubit Protein Assay Kit (Thermo Fisher Scientific, Scoresby, VIC, Australia) according to the manufacturer’s instructions using 10 μL of culture supernatant. Matrix-matched calibration curves were prepared in the presence of halogenated nitrophenols to account for potential fluorescence interference, as previously described [1].

### 2.5. Intracellular Enzyme Assays

#### 2.5.1. Preparation of Intracellular Protein Extracts

Fungal biomass was harvested by centrifugation at 10,000× *g* for 10 min at 4 °C and washed thrice with 50 mM potassium phosphate buffer (pH 7.4) at 4 °C to remove residual medium. Approximately 100 mg of biomass was transferred to screw-cap bead-beating tubes containing the bead matrix supplied with Quick-DNA Fungal/Bacterial Miniprep Kit (Zymo Research, Chatswood, NSW, Australia). Cells were broken by bead-beating (2 × 60 s 6.5 m^−s^), and lysates were clarified by centrifugation at 10,000× *g* for 10 min at 4 °C. The supernatant was collected as the intracellular protein extract [22].

#### 2.5.2. Glutathione S-Transferase (GST) Activity Assay

Glutathione S-transferase (GST) activity was determined using a commercial assay kit (Sigma-Alrich, Cat. No. CS0410) following manufacturer instructions. The assay measures GST-catalyzed conjugation of reduced glutathione (GSH) to 1-chloro-2,4-dinitrobenzene (CDNB), monitored as an increase in absorbance at 340 nm [32]. Intracellular protein extracts were used directly in the assay. Reaction rates were calculated from the linear portion of the absorbance trace. Extracts were prepared from fungal biomass grown under contaminant-free conditions to assess baseline GST activity.

### 2.6. Extracellular Enzyme Activity Under Contaminant and Controls Conditions

To assess the effect of halogenated nitrophenols on enzyme production, *C. fumago* and *Curvularia* sp. were cultured in 100 mL liquid medium in the presence and absence of 2-chloro-4-nitrophenol (2C4NP; 0.3 mM) or 5 fluoro-2-nitrophenol (5F2NP) under the conditions described above. Contaminant-free cultures served as bio-controls to establish baseline extracellular enzyme activity. Crude culture supernatants were collected at defined time points and analyzed for extracellular oxidative enzyme activity, including chloroperoxidase (CPO/VCPO), laccase, manganese peroxidase (MnP), and lignin peroxidase (LiP), using the assays described in Section 2.4. To distinguish enzyme-dependent activity from non-enzymatic background, untreated and heat-denatured supernatants were analysed in parallel. Heat denaturation was performed by incubating aliquots at 100 °C for 20 min to inactivate enzyme activity [33], followed by cooling to 25 °C prior to analysis. For CPO/VCPO assays, heat-denatured supernatants were used as a reference control to correct for non-enzymatic iodide and background absorbance at 350 nm. The acidic assay conditions (pH 2.75) minimized interference form the nitrophenol-derived colour, which is suppressed under acidic conditions [34]. For laccase, MnP, and LiP assays, enzyme-dependent activity was determined by comparing reaction rates between untreated and heat-denatured supernatants under identical assay conditions.

### 2.7. Direct Transformation of Halogenated Nitrophenols by Purified Enzymes

The ability of purified extracellular oxidative enzymes to directly transform halogenated nitrophenols was evaluated to determine whether enzymatic activity alone was sufficient to initiate substrate transformation in the absence of intact fungal systems [18]. Assays were conducted in 4 mL quartz cuvettes with a working volume of 3.0 mL. Substrates (2C4NP and 5F2NP) were added to a final concentration of 0.3 mM. Absorbance at 420 nm was monitored over a 30 min at 5 min intervals at 25 °C. All conditions were prepared in triplicate (*n* = 3), and control reactions lacking enzymes were included to account for non-enzymatic changes in absorbance. These experiments were designed to isolate extracellular oxidative capacity independent of cellular uptake or intracellular metabolism. To evaluate whether inhibition of cytochrome P450 activity influenced extracellular oxidative enzymes profiles, crude supernatant from ABT-treated cultures were analyzed in parallel. Enzyme activities (CPO/VCPO, laccase, MnP, and LiP) were quantified using the same assay conditions described above, enabling direct comparison between inhibitor-treated and untreated systems.

#### 2.7.1. Chloroperoxidase (CPO) Assay

CPO reactions were performed in potassium phosphate buffer (pH 5.5). Purified chloroperoxidase from *C. fumago* (Sigma-Aldrich) was added at 12 μL per reaction from a 1250 U mL^−1^ stock solution. Reactions were initiated by the addition of 20 μL of hydrogen peroxide (H_2_O_2_) with gentle mixing. To maintain oxidate availability, a second 20 μL aliquot of H_2_O_2_ was added after 15 min [18]. Absorbance at 420 nm was recorded at 5 min intervals over 30 min at 25 °C.

#### 2.7.2. Laccase Assay

Laccase reactions were performed in 100 mM phosphate-citrate buffer (pH 5.5) under conditions matched to the CPO experiments to enable direct comparison between enzyme systems. Each reaction contained substrate (0.3 mM), buffer, and purified laccase from *Trametes versicolor* (10 μL, 20 U working solution) to a final volume of 3.0 mL [27]. No redox mediator or external oxidant was added. Absorbance at 420 nm was monitored at 5 min intervals over 30 min. Control reactions lacking enzymes were included.

### 2.8. Genomic Sequencing and Pfam-Based Functional Annotation

#### 2.8.1. DNA Extraction, Sequencing and Genome Assembly

Genomic DNA was extracted from *C. fumago* and *Curvularia* sp. grown in malt extract broth using Quick-DNA Fungal/Bacterial Miniprep Kit (Zymo Research, Chatswood, NSW, Australia) following the manufacturer protocols with minor modifications to improve fungal biomass disruption. These modifications included repeated freeze–thaw cycles in liquid nitrogen and mechanical agitation where required. Long-read sequencing libraries were prepared using the Oxford Nanopore Native Barcoding Kit (SQK-NBD114) and sequenced on a MinION Mk1D device (MinKNOW v25.05.14) using two R10.4.1 flow cells (FLO-MIN114) [35]. Super-accurate basecalling was performed live with Dorado v7.9.8 (super-accurate model r10.4.1_e8.2_400bps_sup@v5.0.0) at a minimum quality score of Q10, resulting in median Q scores of Q24–Q26 of passed reads (41–59% of reads > Q25; N50 2380–4130). The passed reads were concatenated and quality filtered with Filtlong v0.2.1 to remove <1 kb reads and to retain the top 95% of bases by quality. Filtered reads were assembled *de novo* using Flye v2.9.6-b1802cg) (-nano-hq) and assembly subsequently polished with Medaka (v2.1.1), with all parameters set to default, including Flye’s haplotype-collapsing mode [36,37]. Assembly statistics, including total assembly size, contig number, and N50, were obtained from the final Flye assemblies. Genome completeness was subsequently evaluated using BUSCO v5.6.0 as described below.

#### 2.8.2. Genome Completeness Assessment

Genome completeness was evaluated using BUSCO v5.6.0 [38] under genome mode lineage-appropriate fungal datasets. The proportion of complete single copy orthologs was used as the primary indicator of assembly quality and annotation suitability.

#### 2.8.3. Pfam-Based Functional Annotation of Oxidative and Dehalogenation Enzymes

Predicted protein sequences from each assembled genome were annotated using HMMER v3.4 [39] against the Pfam-A database (release 35) [40]. Annotation focused on oxidative enzyme families implicated in oxidative transformation of halogenated contaminants, including vanadium-dependent halo-peroxidase-families (PF01328), heme peroxidases (PF00141), and multicopper oxidases characterized by Cu oxidase domains (PF03098, PF00394, PF07731, PF07732). Cytochrome P450 monooxygenases (PF00067) and glutathione S-transferases (GSTs: PF00043, PF13417) were additionally annotated to assess intracellular oxidative and conjugative detoxification capacity. Vanadium-dependent halo-peroxidase-family proteins (PF01328) were identified in both *C. fumago* and *Curvularia* sp. using HMM searches against the PFAM database [40]. To distinguish canonical *Curvularia*-type chloroperoxidases from divergent halo-peroxidase-family paralogs, proteomes were additionally queried using the biochemically characterized *Curvularia inaequalis* VCPO sequence (UniProt P49053) via BLASTp analysis. This confirmed a near-identical homolog in *Curvularia* sp., while *C. fumago* encoded multiple divergent PF01328-family paralogs. Pfam annotation was used to determine genomic encoding potential for oxidative enzyme families within each genome. These analyses assess predicted domain presence only and do not imply enzyme expression, catalytic activity, or pathway completeness.

#### 2.8.4. Identification of Fluoride Export (Fex-like) Transporters

Putative fluoride export proteins (FEX-link transporters) were identified in *C. fumago* and *Curvularia* sp. using homology-based approach. Predicted proteomes were queried using BLASTp against characterized fungal fluoride export proteins, including FEX1 homologs for *Neurospora crassa* and *Candida albicans* [41]. Candidate sequences were evaluated based on sequence similarity and conserved domain architecture identified through InterProScan annotation and compared with reported characteristics of fungal FEX transporters [42].

### 2.9. Statistical Analysis

All experiments were performed with a minimum of three biological replicates (*n* > 3) per condition. Data are represented as mean ± standard deviation (SD) unless otherwise stated. Statistical analyses were performed using RStudio (v. 2024.12.0+467). For comparisons between fungal treatments and the corresponding control at each time point, two-tailed Welch’s *t*-tests were applied. In addition to *p*-values, effect sizes were calculated as Hedges’ *g* (bias-corrected standardized mean difference) with 95% confidence intervals. Positive Hedges’ *g* values indicate higher activity in the treatment relative to the control, whereas negative values indicate lower activity. Statistical significance was defined as *p* < 0.05. To assess the robustness of the observed patterns and account for potential deviations from normality, non-parametric analyses were also performed. These included Kruskal–Wallis test followed by pairwise Wilcoxon rank-sum test with Benjamini–Hochberg false discovery rate (FDR) correction applied to account for multiple comparisons. Non-parametric analyses produced equivalent outcomes to the parametric test, supporting the robustness of the reported findings. Because experiments were conducted using biological triplicates effect sizes (Hedges’ *g*) and 95% confidence intervals were reported alongside significant testing to aide interpretation of the magnitude and biological relevance of observed differences.

## 3. Results and Discussion

### 3.1. Effect of Media Composition and Cofactor Availability on Halogenated Nitrophenol Transformation

Halogenated nitrophenols were used as a model system to investigate transformation of halogenated contaminants under controlled, growth-constraining conditions. Previous work demonstrated that *C. fumago* and *Curvularia* sp. can degrade these compounds in minimal media, including at concentrations that inhibit fungal growth, indicating that transformation is not strictly coupled to biomass accumulation but instead reflects oxidative processing within the intact fungal system [1]. To examine how environmental constraints influence this behavior, cultures were grown under defined conditions with systematic variation in nutrient availability and cofactor composition. Glucose concentrations were adjusted to represent carbon-replete and carbon-limited conditions, while vanadate supplementation was included to probe the contribution of vanadium-dependent haloperoxidase activity. Under these conditions, both fungal systems exhibited measurable transformation of halogenated nitrophenols across treatments (Table 2). However, transformation rates and extents varied significantly with changes in nutrient availability and cofactor composition, indicating that environmental constraints play a key role in modulating transformation behavior.

Cofactor availability further distinguished the behavior of the two fungal systems. *C. fumago* retained transformation activity across all tested conditions, whereas *Curvularia* sp. exhibited strict cofactor dependence. No measurable transformation of 2C4NP was observed under any condition in *Curvularia* sp., while 5F2NP transformation (81.9%) occurred only in the presence of Na_3_VO_4_ under nutrient-replete conditions (Table 2). No activity was detected under Fe^2+^ or reduced composition conditions, indicating that both cofactor availability and overall nutrient context are required to support transformation in this system. This observation aligns with studies demonstrating that fungal oxidative enzymes systems are highly dependent on metal cofactors, which affect catalytic activity, enzyme stability, and overall transformation efficiency [43]. This cofactor dependence is consistent with previous studies on *Curvularia inaequalis* where growth in the absence of vanadate resulted in the secretion of an inactive apo-form of chloroperoxidase, while the addition of Na_3_VO_4_ restored halogenation activity [19]. These findings indicate that vanadate is required for activation of functional chloroperoxidase, and that enzyme production alone is insufficient without appropriate cofactor incorporation. Although haloperoxidase are traditionally associated with halogenation reactions, they also generate highly reactive oxidative intermediates capable of modifying aromatic substrates and initiating transformation pathways that may ultimately facilitate dehalogenation and downstream detoxification processes [17]. The strict Na_3_VO_4_ dependence observed in the present study suggests that 5F2NP transformation in *Curvularia* sp. is mediated by vanadium-dependent oxidative enzymes requiring active cofactor incorporation to achieve catalytic function. However, previous studies using minimal media supplemented with trace elements have reported transformation of both 2C4NP and 5F2NP in *Curvularia* sp. in the absence of explicitly added Na_3_VO_4_, indicating that background cofactor availability or alternative metabolic pathways may support transformation under those conditions [1]. Taken together, these findings demonstrate clear substrate-specific transformation behavior and strong dependence on the biochemical environment. Transformation in *Curvularia* sp. is highly sensitive to cofactor availability, whereas *C. fumago* exhibits more robust activity across conditions, supporting the presence of distinct system-level mechanisms governing halogenated nitrophenol transformation. These patterns cannot be resolved from transformation data alone and require further investigation at the enzyme and genomic level [21].

### 3.2. Crude Supernatant Transformation Capacity

The transformation behavior observed in Section 3.1 suggests that halogenated nitrophenol transformation was not directly linked to fungal growth and instead reflected extracellular oxidative processes operating under defined biochemical conditions. Transformation occurred under apparent stationary-phase conditions, where little to no measurable biomass increase was observed, consistent with the previous reports in these systems [1]. To determine whether extracellular components alone retained transformation capacity, crude cell-free supernatants were collected from cultures exhibiting active whole-cell transformation and evaluated in the absence of fungal biomass. Supernatants were collected from *C. fumago* cultures grown under Fe^2+^ containing conditions and from *Curvularia* sp. cultures grown under Na_3_VO_4_ supplemented conditions, corresponding to the same conditions that supported whole-culture transformation in Section 3.1. In contrast, supernatants derived from conditions lacking whole-culture transformation, including *Curvularia* sp. grown under Fe^2+^ conditions, did not exhibit measurable extracellular activity. These findings suggest that transformation capacity is strongly influenced by the biochemical environment in which the fungal systems are generated.

**Figure 1:**
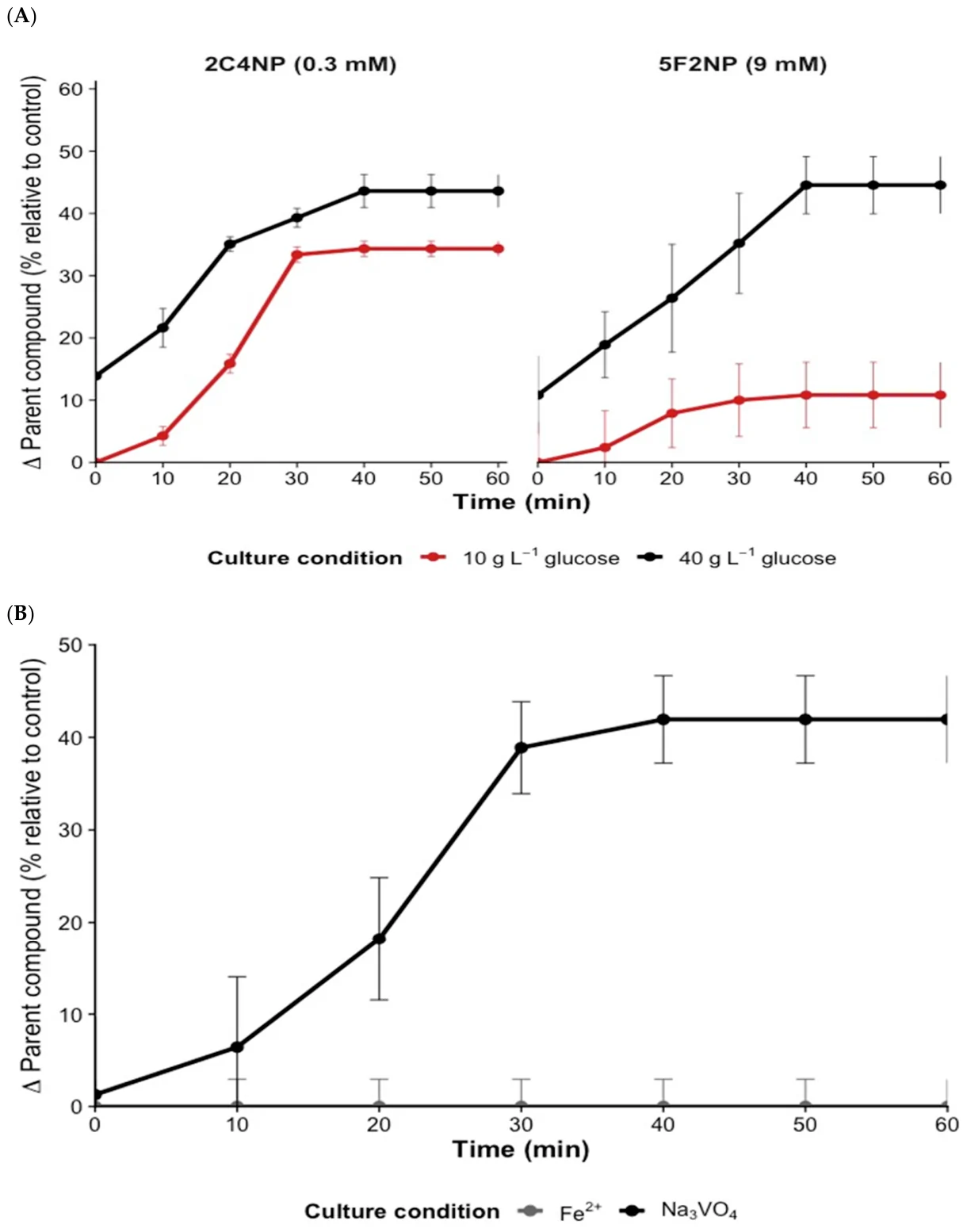
Crude supernatant activity mirrors whole-culture transformation patterns in *C. fumago* and *Curvularia* sp. (**A**) Parent compound depletion of 2-chloro-4-nitrophenol (2C4NP; 0.3 mM) and 5-fluoro-2-nitrophenol (5F2NP; 9 mM) by 56 h *C. fumago* crude supernatants collected from cultures grown under 40 g L^−1^ and 10 g L^−1^ glucose condition. (**B**) Parent compound depletion of 5F2NP (0.3 mM) by 72 h *Curvularia* sp. crude supernatants collected from Fe^2+^ containing and Na_3_VO_4_ supplemented cultures. Depletion was calculated relative to substrate-only controls at each time points. Values represent mean ± SD (*n* = 3).

Crude culture supernatants retained substantial transformation capacity in the absence of fungal biomass (Figure 1). Supernatants derived from *C. fumago* cultures achieved rapid depletion of both 2C4NP and 5F2NP, with approximately 40–45% reduction occurring within 35–45 minus before reaching a plateau (Figure 1A). Similarly, supernatants collected from *Curvularia* sp. cultures grown under Na_3_VO_4_ supplemented conditions achieved ~42% depletion of 5F2NP within 35 min, whereas no measurable activity was observed in supernatants derived from Fe^2+^ conditions (Figure 1B). The correspondence between whole-culture and supernatant behavior demonstrates that extracellular transformation capacity is retained only under biochemical conditions that support active fungal transformation. The rapid initial depletion followed by consistent plateau across systems suggests that extracellular activity becomes system-limited over time, potentially reflecting constraints in cofactor availability, redox cycling capacity, or depletion of reactive intermediates required to sustain catalytic turnover [21,44]. Together, these findings indicate that extracellular transformation depends not only on enzyme presence, but also on the broader biochemical environment in which the oxidative system is generated and maintained.

### 3.3. Purified Enzyme Activity

Because *C. fumago* and *Curvularia* sp. were selected based on their reported haloperoxidase systems, purified chloroperoxidase (CPO) was evaluated to determine whether the principal extracellular oxidase associated with these fungi could independently reproduce the transformation observed in whole cultures. In addition, purified laccases were included as a representative fungal oxidative enzyme because laccases are among the most extensively studied extracellular catalyst implicated in xenobiotic transformation [9,45]. Commercially available purified chloroperoxidase from *C. fumago* and purified laccase from *Trametes versicolor* were tested against both 2C4NP and 5F2NP under assay conditions matched to the extracellular transformation experiments. Neither purified enzyme produced measurable transformation of 5F2NP, and only minimal depletion (~4%) of 2C4NP was observed over the assay period. Laccase did not induce detectable transformation of either substrate. These findings indicate that individual extracellular oxidases, including both chloroperoxidase and laccase, are insufficient to reproduce the transformation observed in intact fungal systems or crude extracellular fractions.

The discrepancy between active crude supernatants and inactive purified enzymes suggested that halogenated nitrophenol transformation cannot be explained by the activity of a single extracellular oxidase. Instead, the results are consistent with the involvement of multiple extracellular enzymes, cofactors, and the redox-active components acting within the native extracellular environment. The specific interactions among these components were not resolved in the present study. Similar behavior has between reported in other fungal oxidative systems, where extracellular transformation depends on mediator compounds, coupled redox chemistry, and interacting enzyme networks rather than isolated catalytic activity [9,21]. Chloroperoxidase from *C. fumago* is a well-characterized heme enzyme capable of halogenation, oxygen transfer, and oxidative dehalogenation reactions involving halogenated phenols and fluorinated substrates [16,17,18,26,46]. Similarly, fungal laccases are widely reported to oxidize phenolic and halogenated aromatic compounds [12]. However, laccase-mediated transformation is strongly influenced by substrate structure and reaction context, with electron withdrawing substituents such as nitro groups reducing susceptibility to direct oxidation [13]. In addition, laccase activity frequently depends on mediator systems or complex extracellular enzyme mixtures, and loss of catalytic efficiency during transformation of substituted phenols has been widely reported [12,13].

Together, these findings support a model in which halogenated nitrophenol transformation depends on coordinated system-level processes rather than the activity of isolated extracellular enzymes. These findings have important implications for field-scale implementation, as they suggest that maintaining intact fungal biomass and coordinated metabolic activity is likely to be more effective than deploying isolated oxidative enzymes. Consequently, engineering fungal biofilms, immobilized mycelial systems, or packed-bed bioreactors may provide more suitable platforms for translating these processes into environmental remediation applications. The environmental significance of this coordinated transformation extends beyond removal of the parent compound. In our previous study using the same fungal species and halogenated nitrophenol substrates, fungal transformation was associated with reduced toxicity of the parent compounds and the formation of less toxic transformation products [1]. Although comprehensive metabolite identification was beyond the scope of the present study, these findings support the interpretation that the coordinated fungal processes described here contribute to contaminant detoxification rather than simple parent compound depletion. Future studies employing high-resolution metabolomic approaches will be valuable for resolving intermediate transformation products and reconstructing complete transformation pathways. However, while the biochemical experiments demonstrate the requirement for interacting oxidative and detoxification processes, they do not identify the specific enzymatic systems responsible for the observed transformation or explain the distinct species- and cofactor-dependent behaviors observed experimentally. Genome-resolved analysis was therefore undertaken to determine the oxidative and detoxification capacity present in each organism and to identify mechanistic differences potentially underlying the observed transformation patterns.

### 3.4. Genome-Resolved Identification of Candidate Enzyme Systems

Genome assemblies for *Curvularia* sp. and *C. fumago* met quality criteria for downstream annotation analysis (Table S1). Comparative functional annotation was subsequently performed to identify oxidative and detoxification enzyme families potentially contributing to halogenated nitrophenol transformation. Genome annotation provides a framework for identifying candidate biodegradation pathways but does not demonstrate enzyme expression or catalytic function. Therefore, genome-resolved analyses were interpreted alongside biochemical assays and transformation experiments to relate predicted metabolic potential to experimentally observe phenotypes [47].

**Figure 2:**
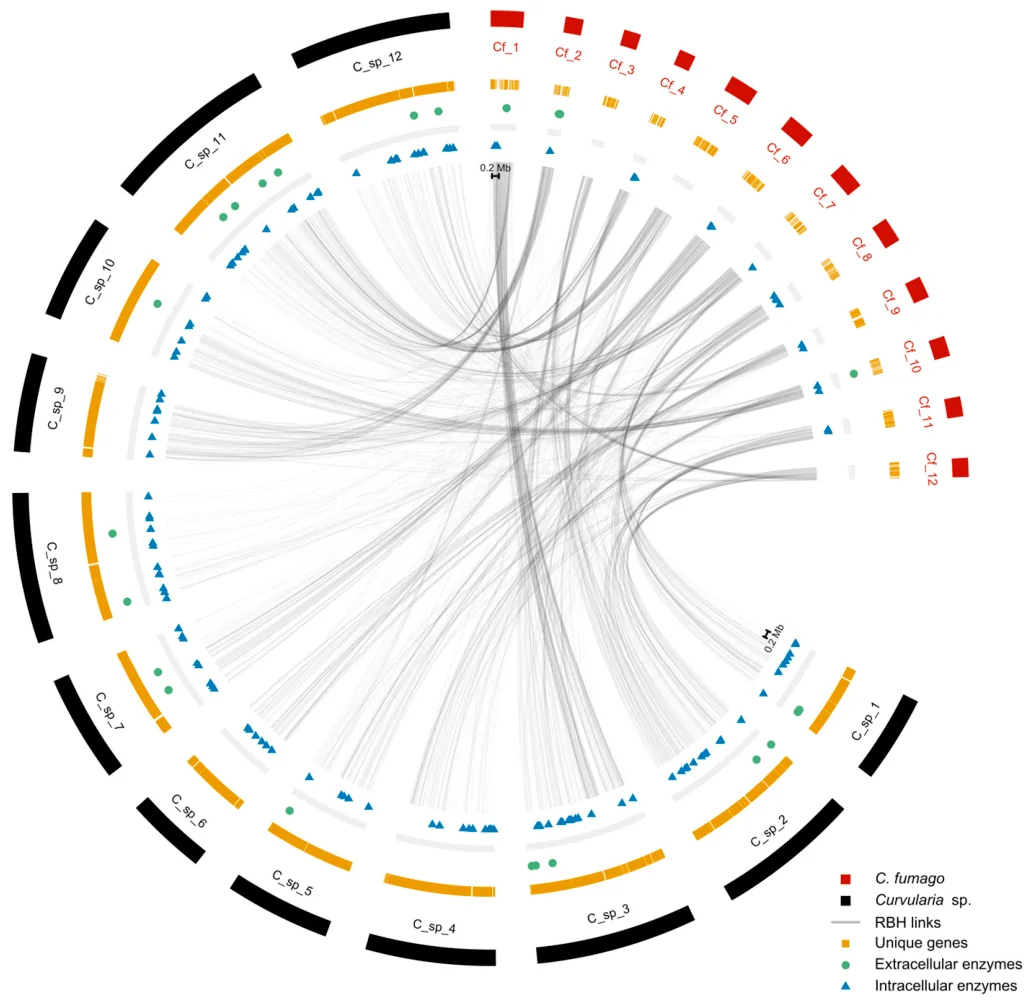
Top 12 contigs from *C. fumago* (red) and *Curvularia* sp. (black). Grey links indicated reciprocal best-hits (RBH) orthologs between genomes. Orange tickets indicate genes lacking RBH orthologs in the opposing genome. Green circles mark extracellular oxidative enzymes, and blue triangles mark intracellular detoxification enzymes.

Genome-resolved analysis of *Curvularia* sp. and *C. fumago* identified multiple enzyme families associated with oxidative transformation and detoxification of halogenated compounds, including heme peroxidases, halo-peroxidases, multicopper oxidases, cytochrome P450 monooxygenases, and glutathione-dependent systems [48]. Comparative analysis integrating orthologous relationships with functional annotation (Figure 2) revealed a conserved intracellular detoxification core shared between species, alongside divergence in extracellular oxidative enzyme systems.

Genome annotation identified genes encoding extracellular oxidative enzymes that differed substantially between species. Both genomes contained genes encoding heme-dependent-chloroperoxidases (PF01328), while *Curvularia* sp. additionally contained genes encoding a putative vanadium-dependent haloperoxidase consistent with previously characterized VCPO systems [19,20]. Heme chloroperoxidases from *C. fumago* are known to catalyze halogenation and oxidative dehalogenation reactions, including transformation of halogenated phenols such as 2,4,6-trihalophenols under acidic conditions [16,26]. These enzymes also exhibit substrate-dependent inhibition in the presence of fluoride, which can compete at the active site and reduce catalytic efficiency [26]. In contrast, vanadium-dependent haloperoxidases operate via a distinct catalytic mechanism in which the vanadate cofactor remains in a stable oxidation state throughout the reaction cycle [20,49]. This removes the requirement for metal centered redox cycling and is associated with stable catalytic behavior under defined conditions [49,50]. Although direct evidence for their role in pollutant transformation remains limited, their catalytic properties suggest potential for sustained extracellular oxidative activity under defined conditions.

Complementing these extracellular systems, both genomes contained genes encoding cytochrome P450 monooxygenases, glutathione S-transferases (GST), and membrane associated transport proteins, indicating the coding potential for a conserved intracellular detoxification system. Cytochrome P450 enzymes have been implicated in the transformation of chlorinated compounds, including trichloroethylene (TCE), where they facilitate intracellular oxidation of xenobiotics [11]. Glutathione S-transferases provide a subsequent detoxification step through conjugation of electrophilic intermediates, supporting intracellular stabilization and processing of transformed compounds [48]. In addition to these enzymatic systems, homology-based analysis identified putative fluoride export proteins (FEX-like transporters) in both *C. fumago* and *Curvularia* sp., with strong similarity to characterized fungal fluoride exporters [41]. Predicted multi-pass transmembrane topology is consistent with the conserved pore-forming architecture described for FEX proteins, supporting their role in transmembrane fluoride transport [42]. These proteins are known to mediate fluoride efflux and confer tolerance to intracellular fluoride accumulation [41,42] which is particularly relevant in the context of fluorinated substrates where intracellular fluoride may otherwise inhibit cellular processes [41]. Together, these findings support a model in which transformation is mediated by coordinated multi-enzyme systems, with extracellular enzymes initiating substrate oxidation and intracellular systems facilitating subsequent detoxification and processing [11,48]. Although both fungi encode a conserved intracellular detoxification framework comprising cytochrome P450 monooxygenases, glutathione S transferases, membrane-associated transporters, and putative FEX-like fluoride exporters, they differed substantially in their predicted extracellular oxidative repertoires. These genomic differences are consistent with the experimental observations, where *C. fumago* maintained transformation across a broad range of nutrient and cofactor conditions, whereas *Curvularia* sp. exhibited a more specialized phenotype dependent upon Na_3_VO_4_ supplementation. The broader extracellular oxidative repertoire predicted for *C. fumago* may therefore provide greater functional flexibility under variable environmental conditions, while the condition-dependent oxidative system of *Curvularia* sp. may enable efficient transformation only when appropriate cofactors are available. From a bioremediation perspective, these findings suggest that extracellular oxidative diversity, rather than intracellular detoxification capacity, is likely to be a key determinant of fungal performance across different contaminant environmental conditions. However successful environmental applications will likely depend on maintaining conditions that support these coordinated oxidative systems, including appropriate nutrient availability, oxygen transfer, cofactor supply, and long-term fungal viability. Evaluating the stability and resilience of these integrated metabolic systems under continuous operation and environmentally variable conditions therefore represents an important direction for future research.

However, while these analyses define the coding potential and enzymatic repertoire present within each fungal genome, they do not establish which genes were actively expressed under experimental conditions. In the present study, extracellular enzyme activities (CPO/VCPO), laccase, LiP, and MnP), GST activity, and cytochrome P450 inhibition were evaluated experimentally, providing functional evidence for oxidative and detoxification processes. Nevertheless, future transcriptomic, proteomic and targeted RT-qPCR analyses will be valuable for directly linking individual gene expression with enzyme production and halogenated nitrophenol biotransformation.

### 3.5. Genome Architecture and Enzyme Distribution in Curvularia sp.

**Figure 3:**
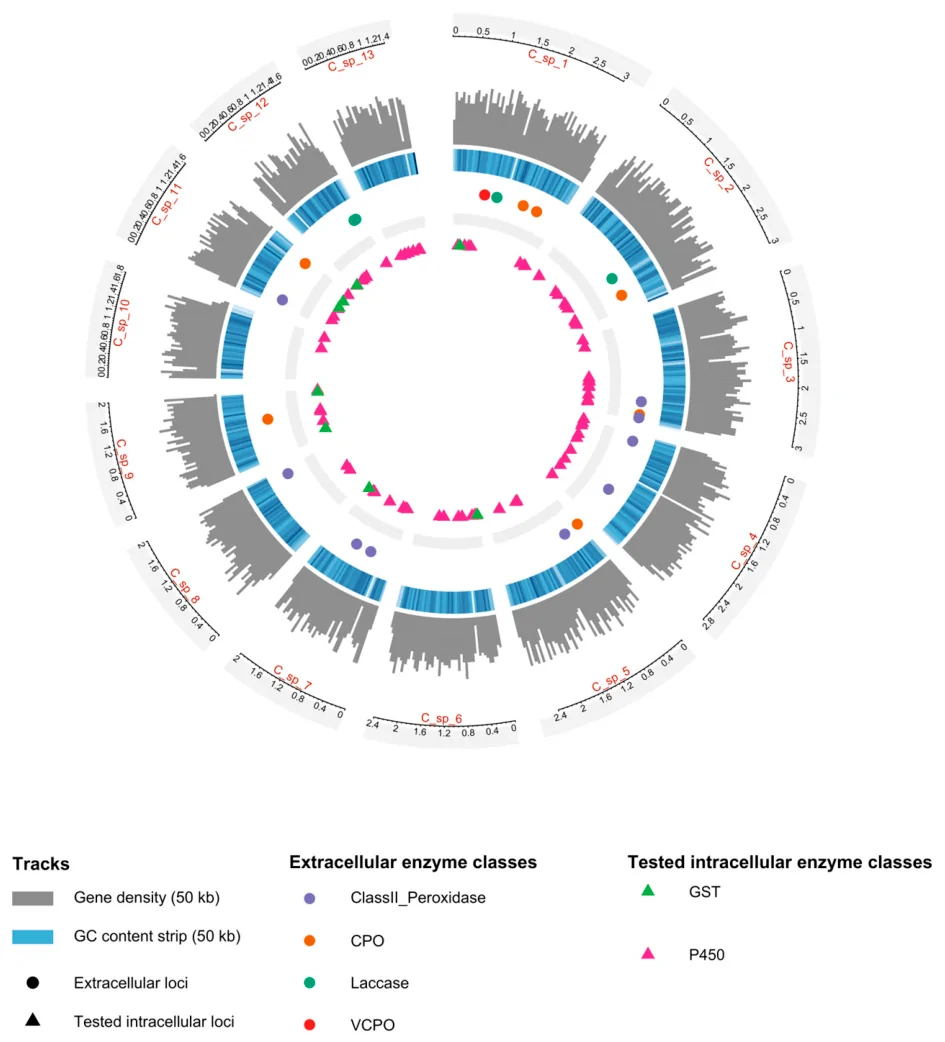
Genome architecture and enzyme distribution in *Curvularia* sp. Circular map of the 13 largest contigs. Contigs are ordered by descending size; spatial proximity between contigs does not reflect chromosomal linkage. Tracks show gene density (50 kb windows; grey) and GC content (blue gradient). Extracellular enzyme loci (circles) include laccase, chloroperoxidase (CPO), Class II peroxidases (lignin peroxidase and manganese peroxidase), and vanadium-dependent chloroperoxidase (VCPO). Intracellular loci (triangles) are limited to cytochrome P450 and glutathione S-transferase (GST). Points indicate gene midpoint positions.

Following the identification of distinct extracellular oxidative and conserved intracellular detoxification systems (Section 3.4), genome architecture was examined to determine whether the spatial organization of these enzyme classes provides additional insights into the transformation behavior observed experimentally. Genome-resolved analysis of *Curvularia* sp. revealed that oxidative and detoxification-associated enzyme systems are distributed across multiple contigs rather than being confined to discrete genomic regions (Figure 3). Extracellular oxidative enzymes, including vanadium-dependent halo peroxidases (VCPO), multicopper oxidases and class II peroxidases, were identified across several genomic locations, indicating a dispersed organization of oxidative capacity.

Oxidative and detoxification enzyme families were identified across multiple assembly contigs rather than being restricted to single genomic regions. This pattern is consistent with previous observations that fungal oxidative and detoxification systems, including cytochrome P450 monooxygenases and glutathione S-transferases are frequently encoded by multigene families distributed throughout fungal genomes rather than organized into operon-like structures [51,52]. Intracellular enzyme systems, including cytochrome P450 monooxygenases and glutathione S-transferases, were similarly identified on multiple contigs, consistent with the conserved detoxification capacity described in Section 3.4. The presence of VCPO homologs within this broader distribution is consistent with the observed requirement for vanadate during 5F2NP transformation (Section 3.1) [20]. However, because these analyses are based on draft genome assemblies, chromosomal linkages between loci cannot be resolved. Consequently, the observed distribution across assembly contigs should not be interpreted as evidence of genome-wide dispersal or functional independence. Furthermore, genome annotation reflects encoding potential rather than expression or catalytic activity [48]. Therefore, while the genome architecture is consistent with transformation involving multiple enzyme systems, direct relationships between genomic organization and contaminant transformation cannot be established from these data alone. The condition-dependent behavior observed in *Curvularia* sp. further suggests that transformation depends not only on enzyme presence but also on environmental and cofactor-dependent activation of oxidative capacity [9,20].

### 3.6. Genome Architecture and Enzyme Distribution in Caldariomyces fumago

**Figure 4:**
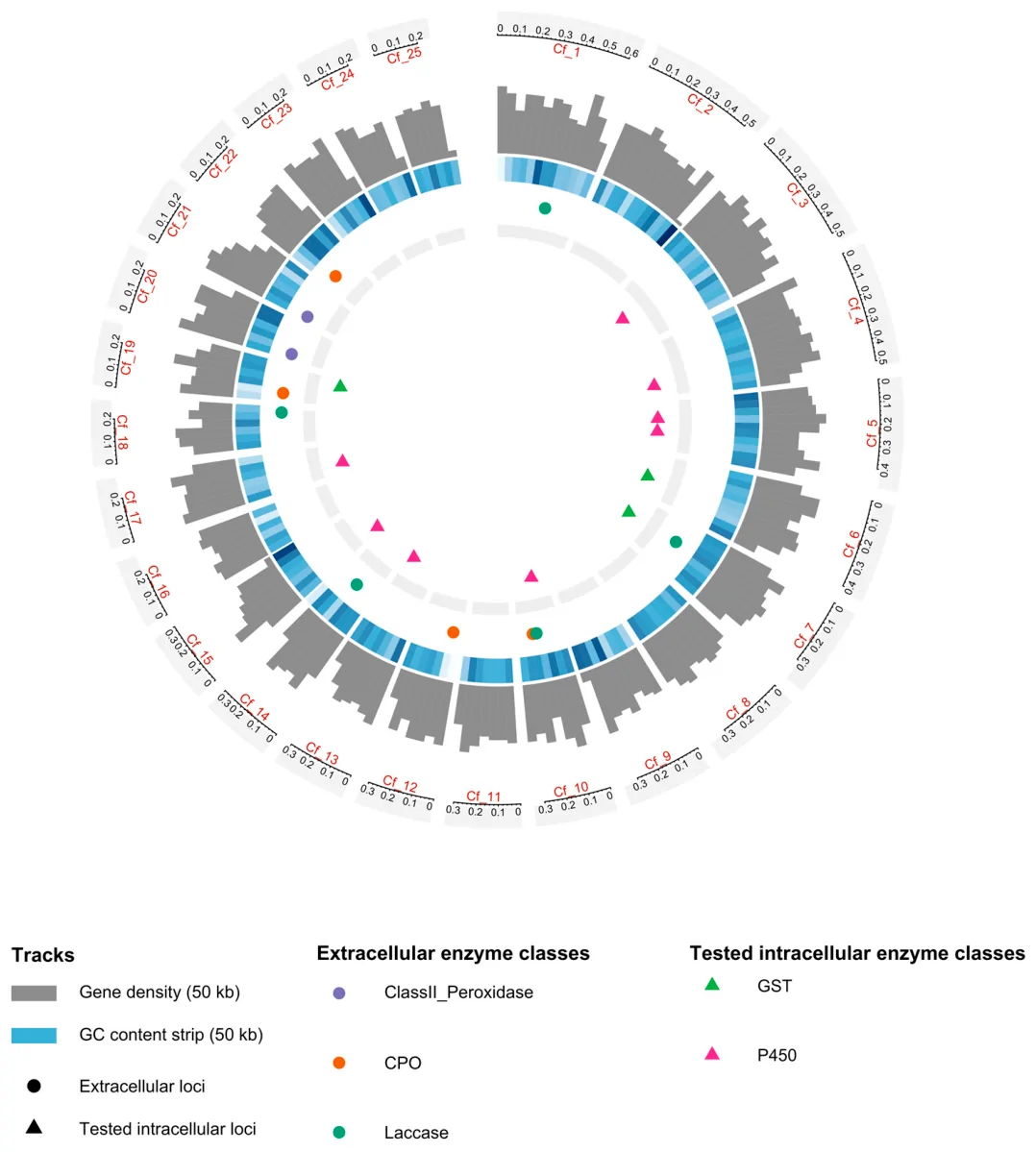
Genomic architecture and enzyme distribution in *C. fumago*. Circular map of the 25 largest contigs. Contigs are ordered by descending size; spatial proximity between contigs does not reflect chromosomal linkage. Tracks show gene density (50 kb windows; grey) and GC content (blue gradient). Extracellular enzyme loci (circles) include laccase, chloroperoxidase (CPO), and Class II peroxidases (lignin peroxidase and manganese peroxidase). Intracellular loci (triangles) are limited to cytochrome P450 and glutathione S-transferase (GST). Points indicate gene midpoint positions.

Building on the distributed enzyme organization observed in *Curvularia* sp. (Section 3.5), the genome architecture in *C. fumago* was examined to determine whether differences in enzyme composition and distribution could account for the more consistent transformation behavior observed experimentally. Genome-resolved analysis of *C. fumago* revealed a similarly distributed organization of oxidative and detoxification-associated enzyme systems across multiple contigs (Figure 4). However, in contrast to *Curvularia* sp., *C. fumago* exhibited a broader representation of extracellular oxidative enzymes, including multiple chloroperoxidase homologs, multicopper oxidases, and class II peroxidases distributed throughout the genome.

This expanded oxidative repertoire is consistent with the well-characterized catalytic versatility of heme-dependent chloroperoxidases, which catalyze halogenation, oxygen transfer, and oxidative dehalogenation reactions across a range of halogenated substrates [16,18,26]. Chloroperoxidase from *C. fumago* has been shown to oxidize phenolic and halogenated compounds under acidic conditions, support its role in broad substrate transformation [17,18]. In contrast to the vanadate-dependent systems observed in *Curvularia* sp., these heme-dependent enzymes do not require alternative metal cofactors for activation, which is consistent with the sustained transformation of both 2C4NP and 5F2NP across all tested conditions (Section 3.1). However, as with *Curvularia* sp., genome annotation reflects encoding potential rather than expression or catalytic activity, and therefore direct linkage between gene distribution and functional output remains inferential [48]. Intracellular detoxification systems, including cytochrome P450 monooxygenases and glutathione S-transferases were similarly well represented and distributed throughout the genome, indicating a conserved capacity for downstream detoxification and metabolite processing. This organization mirrors that observed in *Curvularia* sp., reinforcing the interpretation that differences in transformation behavior is not driven by intracellular capacity but instead reflect variation in extracellular oxidative systems. Taken together, these findings support a model in which the broader extracellular oxidative enzyme complement in *C. fumago* highlights a more robust and less conditionally constrained transformation system.

### 3.7. Extracellular Enzyme Induction Under Contaminant Exposure

**Figure 5:**
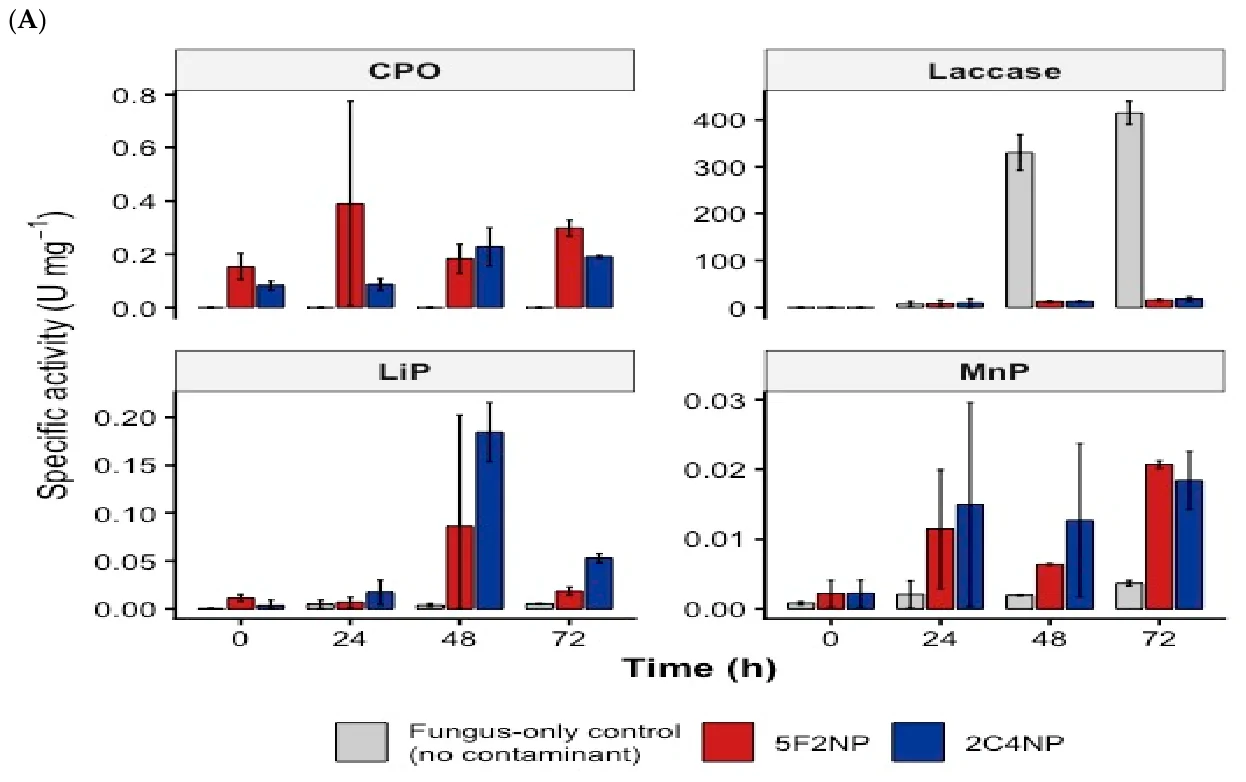

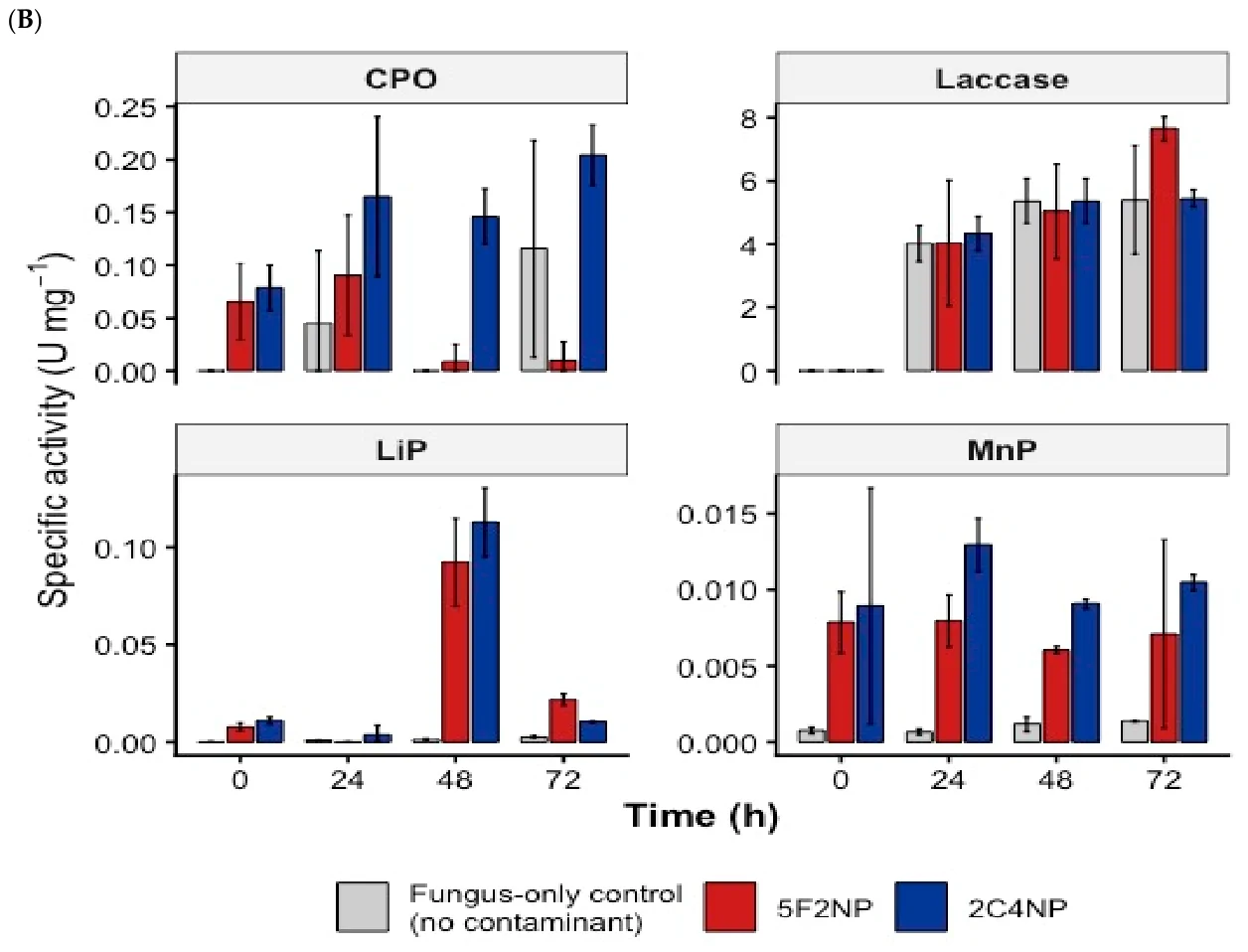
Extracellular enzyme activity profiles under contaminant exposure. (**A**) Specific activity (U mg^−1^ extracellular protein) of chloroperoxidase (CPO/VCPO), laccase, lignin peroxidase (LiP), and manganese peroxidase (MnP) produced by *Curvularia* sp. over 72 h under fungus-only (grey), 5F2NP (red), and 2C4NP (blue) conditions. (**B**) Specific activity (U mg^−1^ extracellular protein) of CPO, laccase, LiP, and MnP produced by *C. fumago* under the same conditions at 56 h. Values represent mean ± SD (n = 3).

Following genome-resolved identification of oxidative enzyme systems (Section 3.4, Section 3.5 and Section 3.6) and the observation that crude supernatants retain transformation capacity whereas purified enzymes do not (Section 3.2), extracellular enzyme activity was quantified to determine whether these systems were present and induced under contaminant exposure conditions (Figure 5). This was assessed as a binary test: (i) whether oxidative enzymes are detectable in crude supernatants, and (ii) whether their activity increases in the response to halogenated nitrophenols. Extracellular activities of CPO/VCPO, laccases, manganese peroxidases (MnP), and lignin peroxidase (LiP) were measured under contaminant (2C4NP, 5F2NP) and contaminant-free (fungus only) conditions (Figure 5A,B). Across both fungal systems, LiP was the only enzyme that consistently exhibited inducible behavior. LiP activity showed a transient increase at 48 h under both substrates, coinciding with the period of maximum transformation observed in Section 3.1 (T_max_ 56–72 h). This pattern is consistent with the established role of lignin-modifying peroxidases in the oxidative transformation of phenolic and halogenated compounds, including chlorinated phenols and substituted aromatics under peroxidase-dependent conditions [53,54,55]. 

In contrast, laccase activity was not induced by contaminant exposure. Although laccase activity dominated across all conditions, it did not increase in response to either substrate or in *Curvularia* sp. and was suppressed under contaminant conditions. This decoupling between laccase activity and transformation indicates that high constitutive oxidative activity alone does not account for substrate transformation. MnP activity remained low and showed no consistent induction. CPO/VCPO activity showed species-dependent behavior. In *C. fumago*, CPO activity was detectable across all conditions and increased under 2C4NP exposure, whereas reduced activity under 5F2NP at early time points is consistent with fluoride-mediated inhibition of chloroperoxidase activity [26]. In *Curvularia* sp., VCPO-associated activity was only observed under Na_3_VO_4_-supported conditions, aligning with the strict cofactor dependence observed in Section 3.1. All enzyme activities were detected in crude supernatants, confirming that oxidative activity is present extracellularly and consistent with transformation observed in crude supernatants (Section 3.2). However, the inability of purified enzymes to reproduce this behavior suggests that transformation does not arise from a single extracellular enzyme but instead reflects coordinated interactions between multiple oxidative enzymes, cofactors, and redox-active components within the extracellular environment [9,21]. Intracellular glutathione S-transferase (GST) activity was detected in both fungi, confirming the presence of downstream detoxification capacity [48]. This was assessed as a binary presence/absence test and supports the existence of intracellular conjugation systems consistent with genome predictions. Inhibition of cytochrome P450 using ABT did not alter transformation kinetics (Section 3.1), indicating that P450 mediated oxidation is not rate-limited under the conditions tested. Taken together, these results demonstrate that extracellular enzyme activity is electively inducible rather than uniformly upregulated, with LiP emerging as the primary contaminant-response enzyme system. Combined with the requirement for crude supernatant activity and the limited activity of purified enzymes, this supports a system-level model in which extracellular oxidative enzymes networks initiate substrate transformation while intracellular processes contribute to the downstream detoxification and metabolite handling.

## 4. Conclusions

This study investigated whether transformation of halogenated nitrophenols by *Caldariomyces fumago* and *Curvularia* sp. could be explained by individual extracellular enzymes or instead required coordinated fungal system-level metabolism. Whole-culture experiments demonstrated clear species, substrate, nutrient, and cofactor-dependent transformation behavior. *C. fumago* retained transformation activity across all tested conditions, whereas *Curvularia* sp. exhibited strict dependence on Na_3_VO_4_ supplementation for 5F2NP transformation. These contrasting responses indicate that contaminant transformation is governed by distinct, condition-dependent enzymatic systems rather than a common transformation pathway. Crude supernatants retained rapid extracellular transformation activity and mirrored whole-culture behavior, while supernatants derived from inactive growth conditions showed no measurable activity. In contrast, purified chloroperoxidase and laccase failed to reproduce the transformation observed in intact cultures or crude extracellular fractions. These findings demonstrate that transformation cannot be explained by individual extracellular enzymes alone and instead depends on coordinated interactions between oxidative enzymes, cofactors, and redox-active components within the native extracellular environment. Genome-resolved analysis supported this interpretation by identifying multiple oxidative and detoxification-associated enzyme systems across both fungi, including chloroperoxidases, multicopper oxidases, cytochrome P450 monooxygenases, glutathione S-transferases, and putative fluoride export proteins. Extracellular enzyme profiling further showed that lignin peroxidase was the only enzyme consistently induced under contaminant exposure, whereas laccase activity remained constitutive and did not correlate with transformation behavior. Collectively, these findings indicate that fungal transformation of halogenated nitrophenols is governed by distinct, condition-dependent enzymatic systems rather than a common transformation pathway. A key strength of this study was the integration of extracellular enzyme assays, intracellular detoxification analyses, and genome-resolved functional annotation, allowing predicted metabolic potential to be interpreted alongside experimentally validated transformation phenotypes. This combination system level approach provides a more comprehensive framework for understanding fungal biodegrading than either biochemical or genomic analyses alone and supports the development of fungal bioremediation strategies that preserve coordinated extracellular and intracellular transformation processes. Future studies should evaluate the performance, stability, and scalability of this integrated fungal system under environmentally relevant operating conditions, including both batch and continuous-flow systems.

## Figures and Tables

**Table 1:** Experimental design for carbon and medium conditions.

Fungus	Medium Condition	Glucose (g L^−1^) Tested
*C. fumago*	Fe^2+^- containing base medium	40, 30, 20, 10
*Curvularia* sp.	Fe^2+^-containing base medium	40, 30, 20, 10
*Curvularia* sp.	Fe^2+^-omitted, Na_3_VO_4_-supplemented medium	40, 30, 20, 10

**Table 2:** Differential effects of carbon availability and cofactor composition on transformation of 2-chloro-4-nitrophenol (2C4NP) and 5-fluoro-2-nitrophenol (5F2NP) by *Caldariomyces fumago* and *Curvularia* sp. under defined media conditions.

Contaminant	Fungus	Growth Conditions	Transformation (%)	tMax (h)
2C4NP	*C. fumago*	40 g L^−1^ glucose (+Fe^2+^)	100	56
2C4NP	*C. fumago*	30–10 g L^−1^ glucose (+Fe^2+^)	82–85.5	72
2C4NP	*Curvularia* sp.	40–10 g L^−1^ glucose (+Fe^2+^)	0	-
2C4NP	*Curvularia* sp.	40 g L^−1^ glucose (+Na_3_VO_4_)	0	-
5F2NP	*C. fumago*	40 g L^−1^ glucose (+Fe ^2+^)	85.3	56
5F2NP	*C. fumago*	30–10 g L^−1^ glucose (+Fe^2+^)	10–25	≤ 32
5F2NP	*Curvularia* sp.	40 g L^−1^ glucose (+Fe^2+^)	0	-
5F2NP	*Curvularia* sp.	40 g L^−1^ glucose (+Na_3_VO_4_)	81.9	72

tMax = time to maximum observed transformation, - = no observed transformation. Glucose concentrations represent proportional reduction in the defined growth medium composition (40–10 g L^−1^ glucose). Fe^2+^ conditions refer to media supplemented with FeSO_4_•H_2_O, while Na_3_VO_4_ conditions refer to sodium orthovanadate supplementation used to support vanadate-dependent chloroperoxidase activity. Transformation (%) represents the maximum observed reduction in parent compound concentrations relative to the initial concentration.

## Data Availability

The original contributions presented in this study are included in the article/Supplementary Material. Further inquiries can be directed to the corresponding author.

## Supplementary Materials

The following supporting information can be downloaded at: https://www.mdpi.com/article/10.3390/jof12070493/s1, Table S1: Assembly and quality metrics for *Curvularia* sp. and *C. fumago* genomes.

## Abbreviations

The following abbreviations are used in this manuscript:

ABT	1-Aminobenzotriazole
BUSCO	Benchmarking Universal Single-Copy Orthologs
CPO	Chloroperoxidase
GST	Glutathione S-transferases
LiP	Lignin peroxidase
MnP	Manganese Peroxidase
PFAM	Protein Families Database
VCPO	Vanadium-dependent chloroperoxidase
2C4NP	2-chloro-4-nitrophenol
5F2N	5-fluoro-2-nitrophenol
FEX	Fluoride Export Protein
GC-MS	Gas Chromatography–Mass Spectrometry
TLC	Thin-Layer Chromatography
HMM	Hidden Markov Model
HMMER	Hidden Markov Model-based analysis software
PDA	Potato Dextrose Agar
SD	Standard Deviation
