# Supplementary material for: Beyond Single Enzymes: System-Level Fungal Transformation of Halogenated Nitrophenols
Source: J Fungi (Basel). 2026 Jul 4;12(7):493. doi: 10.3390/jof12070493 (PMC13413372; doi:10.3390/jof12070493)
Supplement: Supplementary file 1 [file jof-12-00493-s001.zip › jof-4391510-supplementary.pdf]

Table S1. Assembly and quality metrics for *Curvularia* sp. and *C. fumago* genomes. Metrics included assembly contiguity, completeness (BUSCO), GC content, sequencing coverage, and estimated contamination. Both genomes exhibited high completeness, supporting their use for downstream functional analyses.

**Genome**	**Total Mb**	**Number of Reads called (M)**	**Assembly size (Mb)**	**Number of contigs**	**N50 (Mbp)/ L50 (contigs)**	**Longest contig (Mbp)**	**GC content (%)**	**Sequencing coverage**	**BUSCO (%)**	**Estimated contamination (%)**
Curvularia sp.	6080	3.04	30.96	23	3.79/23	3.33	50.23	~185x	C: 99.5, [S:99.5, D:0.1], F:0 M:0.5	12
C. fumago	2540	1.47	47.28	290	4.77/30	1.71	51.08	~110	C: 97.2 [S:95.9, D: 1.3], F: 0.2, M:1.2	2

BUSCO abbreviations: C, Completeness; S, Single-copy; D, Duplicated; F, Fragmented; M, Missing.
